# Applying the vaginal approach to ovarian cystectomy: current evidence and future applications

**DOI:** 10.2144/fsoa-2020-0083

**Published:** 2020-06-26

**Authors:** Nicolas Galazis, Stephanie Mappouridou, Srdjan Saso, Konstantinos Lathouras, Joseph Yazbek

**Affiliations:** 1 West London Gynaecological Cancer Centre, Queen Charlotte’s Hospital, Hammersmith Hospital Campus, Imperial College London, Du Cane Road, London, W12 0NN6 UK

**Keywords:** intraoperative ultrasound, vaginal ovarian cystectomy, vaginal pelvic surgery

We are very pleased that our colleagues have shown interest in our review [1]. Indeed, we do hope that this review will ignite more interest in vaginal adnexal and vaginal natural orifice transluminal endoscopic surgery (vNOTES). We thank the authors for their positive feedback on our work.

In response to the comments raised, the authors are right to point out that bladder injury has been described in vNOTES hysterectomy and not in adnexal surgery, as reported by Baekeladt *et al.* (2019) and this complication was not statistically significant when compared with the conventional total laparoscopic hysterectomy [2]. Moreover, the authors reiterate our opinion that a complication such as bladder injury during vaginal ovarian cystectomy is anatomically very unlikely. We agree that more information will be obtained after the publication of the multicentre prospective complication database which will shed more light to the matter.

Indeed vNOTES does not require abdominal incisions but involves pneumoperitoneum, another factor that could potentially add to postoperative pain. For this reason, we are of the opinion that a simple vaginal ovarian cystectomy with ultrasound guidance as proposed in the review could be a vaginal method that warrants more evaluation. Furthermore, we agree that the vaginal approach in general is promising in terms of postoperative pain and further studies will clarify that in the future.
